# Disparities in the expansion of telemedicine in pediatric specialty care through the COVID-19 pandemic and beyond

**DOI:** 10.1016/j.sipas.2025.100275

**Published:** 2025-02-14

**Authors:** Monalisa Attif Hassan, Jeffrey Fine, Kathleen Doyle, Emily Byrd, Minna M. Wieck

**Affiliations:** 2425 Stockton Blvd., Room 517, Sacramento, CA, USA

**Keywords:** Telemedicine, Telehealth, COVID-19, Pediatric Surgery

## Abstract

**Introduction:**

The COVID-19 pandemic resulted in the rapid expansion of telemedicine, including in specialties traditionally dependent on physical exams, such as pediatric surgery. Trends in its utilization as in-person visits resumed are not well understood, nor is its effect on mitigating disparities related to social determinants of health (SDOH). We hypothesize that telemedicine utilization increased after the pandemic and has remained higher compared to pre-pandemic levels. Additionally, we hypothesize that increased telemedicine use has contributed to lower no-show rates and more equitable access to care.

**Methods:**

A retrospective cohort analysis was conducted of all outpatient visits at a single outpatient pediatric surgery clinic at a quaternary academic center from 01/02/2018 to 10/26/2022. Clinical variables extracted included demographic data, no-show rate (patient did not attend scheduled appointment), and visit type (in person vs telemedicine). Geocoded census data was used to determine SDOH variables such as internet and computer access. A mixed effect logistic regression model was performed to identify which variables were associated with differences in telemedicine usage.

**Results:**

6339 encounters for 2735 patients were analyzed. Odds of presenting to a scheduled telemedicine visit compared to an in-person visit was 0.76 (CI 0.63-0.91, p-value < 0.01). The odds of selecting a telemedicine visit decreased by 34 % for Spanish speakers and 63 % for ‘other’ language speakers compared to English speakers (p-value < 0.01). The odds of choosing a telemedicine visit also decreased by 4 % for every one-unit increase in the probability of having access to the internet (p < 0.01). There was no significant difference in the odds of choosing a telemedicine visit for insurance status, age, distance, or probability of having access to a computer.

**Discussion:**

Telemedicine continues to be utilized at higher rates compared to pre-pandemic levels, but does not reduce no-show rates, which may reflect limits in its clinical utility. It is used less frequently by non-English speakers, which may contribute to ongoing disparities in access to specialty pediatric care.

## Highlights

• Telemedicine use expanded during the pandemic and infrastructure has remained widely available since.
• Although telemedicine infrastructure remains, post-pandemic utilization of telemedicine for ambulatory pediatric specialty care has decreased. This may reflect patient and/or provider preferences and the limitations of telemedicine.
• Demographic differences exist in the use of telemedicine in the ambulatory pediatric specialty care setting. These differences vary before, during, and after state-mandated stay at home orders.

## Introduction

The COVID-19 pandemic resulted in a rapid transformation of healthcare delivery with the expansion of telemedicine [1]. The mandated stay-at-home order in California officially began on March 19, 2020 and ended January 25, 2021, restricting the movement of patients to hospitals and clinical settings while shifting healthcare services to caring for COVID-19 patients in a state of emergency [2,3]. These barriers to in-person care were addressed with synchronous video technology to conduct patient visits, including for pediatric subspecialty providers.

Initially, several barriers existed to offering telemedicine visits: reimbursement, lack of provider time, lack of provider interest, and state regulations. The state-of-emergency invoked by the COVID-19 pandemic eliminated many of these barriers almost instantly: Medicare and Medi-Cal began reimbursement and updated policies allowed patients to conduct visits from their homes in a Health Insurance Portability and Accountability Act (HIPAA) compliant manner [4,5]. In fact, two months into the pandemic, 81 % of primary care practices nationwide were offering telemedicine [1].

Prior to the stay-at-home order, telemedicine visits were offered inconsistently. In the field of pediatric primary care, telemedicine was utilized at a rate of 15 % despite endorsement by the American Association for Pediatrics (AAP) for general pediatric care in 2016 [4]. The AAP cautioned against subspecialty pediatric utilization of telemedicine due to concerns for lack of integrated follow-up, suboptimal care quality, and fragmentation of care [4].

Pediatric patients are a unique patient population and present different telemedicine challenges compared to adult patients. For example, scheduling appointments can be more difficult for pediatric patients due to the need to align both caretaker work requirements and the patient's school schedules. Additionally, children often have limited ability or vocabulary to communicate their symptoms, necessitating a physical exam by a professional for diagnosis. Common pathologies such as inguinal hernias, undescended testicles, or pilonidal disease are diagnoses where the child may not be aware of exactly what is wrong and may not feel comfortable talking to their parent about it. Parents may also have privacy concerns about sending pictures or doing any portion of a physical exam over video, especially for problems in sensitive areas like the groin/perineum and breasts. Lastly, the effort to minimize unnecessary imaging and laboratory tests in children also places more importance on accurate physical examination for diagnoses. Thus, if telemedicine usage is to continue for pediatric care, assessing the impact of telemedicine utilization on patient access to pediatric subspecialty care is essential to ensure equitable and optimal care.

The widespread implementation of telemedicine visits provides an opportunity to assess patient barriers to accessing pediatric specialty care. That is, how does technology interact with social determinants of health (SDOH) in vulnerable patient populations? SDOH are factors which affect daily needs, including safe housing, access to educational, economic, and job opportunities, and access to healthcare services [6,7]. SDOH affects in-person access to care but has also been shown to impact virtual healthcare delivery. Previous studies show disparities in completing a telemedicine visit based on low income, female sex, and identifying as Black or African American [8]. Additionally, pandemic-specific social factors also affected access to telemedicine. For example, many essential workers come from communities of color and could not work from home, limiting the ease of logging into a visit [7]. Understanding telemedicine utilization trends post-pandemic and the ongoing barriers for patients and providers will help optimize effectiveness and minimize persistent disparities in access to care.

The purpose of this study was to assess trends in the utilization of telemedicine visits in pediatric surgical patients before, during, and after the COVID-19 stay-at-home order. We also sought to evaluate disparities in telemedicine utilization by patients for quality improvement purposes. We hypothesized that (1) telemedicine utilization would remain high after the pandemic, (2) increased telemedicine utilization would decrease no-show rates and expand access to care across demographics, and (3) different demographic groups would utilize telemedicine equitably.

## Methods

A retrospective cohort study was conducted at a single quaternary referral pediatric surgical center. Data on every patient encounter at a pediatric surgery clinic between January 2^nd^, 2018 and October 26^th^, 2022 was extracted from the electronic medical record. Using the official stay-at-home order dates of March 19, 2020 and January 25, 2021, data was separated into pre-pandemic (807 days), during pandemic (312 days), and post-pandemic (639 days) periods. Per institutional guidelines, the study was not considered human subjects research and did not require IRB review.

For this clinic, the default modality for all visits during the pandemic was telemedicine unless the attending surgeon specifically requested an in-person visit, usually because a physical exam was essential for diagnosis or operative planning. After the stay-at-home order was lifted, patients were still offered a telemedicine appointment but could choose an in-person visit if they desired. Surgeons could still request an in-person only appointment based on clinical indications.

The primary clinical variables of interest were visit modality (in-person versus telemedicine) and no-show rates. The term ‘no-show’ describes when a patient did not present to a scheduled clinic visit. These variables were compared over time and between different demographic and SDOH-related variables. Demographic data collected for each patient included race/ethnicity, zip code, age, gender, language, insurance type, and diagnosis. This information was available in the electronic medical record. Missing data for ‘ethnicity’ and ‘language’ (N = 2) were coded with the category of ‘unknown/declined’ for analysis. Distance from clinic was measured in driving miles based on the patient's home zip code.

Publicly available United States Census data on patient zip code was used to gather geocoded percent of households with any kind of computer, including smartphone, and percent of households with any kind of internet access including dataplan [9]. The website was utilized as follows: patient zip-code is searched in the Census website and results in a percent. This percent represents the proportion of households in that zip-code with access to a computer or data plan. These proportions were used in the analysis to represent the probability a patient in that zip-code has access to a computer or to the internet. An example of the output is shown in Supplement A.

A mixed effect logistic regression model was performed using SAS 9.4 (SAS Institute Inc. 2014, Cary, NC) to identify which demographic or clinical variables were associated with differences in telemedicine usage. Subject was used as a random effect to account for the correlation between patients who had multiple visits during the study period. Univariate analyses were performed first on demographic and clinical variables of interest which included age, sex, insurance status (private insurance or other), primary language spoken (English, Spanish, or other), race/ethnicity (White/Caucasian, Black/African American, Asian, Hispanic/Latino, other, or unknown/declined to state), miles from a hospital based on zip code, access to a computer based on zip code, and access to the internet based on zip code. A secondary hypothesis looking at associations for completed visits versus no-shows also used a similar multivariable mixed effects logistic regression model with patient ID as a random effect. This model included the same variables of interest but also included the visit type in the model. Finally, a multivariable mixed effect logistic regression model, with patient ID as a random effect, was used to see if time period had an impact on if the patient had a completed visit versus being a no-show after adjusting for other predictors. Hypothesis tests were two-sided and evaluated at a significance level of 0.05.

## Results

A total of 6339 encounters for 2735 patients were analyzed. 2455 visits occurred before the pandemic (21.2 visits/week), 1054 during (23.9 visits/week), and 2830 after (31.1 visits/week). During the entire study period, 32.9 % were telemedicine visits. The highest percentage of telemedicine visits occurred during the pandemic (0.7 % before, 71.1 % during, 46.6 % after). Demographic data of all encounters is detailed in Table 1.

**Table 1:** Univariate mixed effect model: odds of choosing a telemedicine visit by clinical variable. Multivariable mixed effect logistic regression analysis including all predictors of interest: odds of choosing a telemedicine visit.

	Univariate Analysis: Odds of Choosing a Telemedicine Visit [Confidence Interval] (p-value)	Multivariable Analysis: Odds of Choosing a Telemedicine Visit [Confidence Interval] (p-value)
Increasing Age	1.16.01 – 1.03] (p = 0.01)	1.01[1.0-1.03] (p < 0.01)
Female	1.31 [1.13 – 1.52] (p < 0.01)	1.33 [1.14-1.54] (p < 0.01)
Spanish Speaking	0.75 [0.26 – 0.66] (p = 0.04)	0.73 [0.54-0.99] (p < 0.05)
Other Language	0.42 [0.57 – 0.98] (p < 0.01)	0.41 [0.26-0.66] (p < 0.01)
Public Insurance	0.99 [0.828 – 1.11] (p = 0.57)	1.01 [0.86-1.17] (p = 0.93)
Race/Ethnicity		
Asian	0.86 [0.61 – 1.18] (p = 0.36)	0.97 [0.70-1.34] (p = 0.85)
Black or African American	0.75 [0.57 – 1.08] (p = 0.14)	0.81 [0.58-1.12] (p = 0.19)
Latinx/Hispanic	0.912 [0.762 – 1.09] (p = 0.32)	0.95 [0.78-1.2] (p = 0.64)
Other	1.01 [0.81 – 1.26] (p = 0.95)	1.10 [0.87-1.38] (p = 0.41)
Unknown/Declined	1.43 [1.05 – 1.93] (p = 0.02)	1.45 [1.1-1.97] (p = 0.02)
Distance From Clinic	1.00 [1.00 – 1.00] (p = 0.51)	1.0 [1.0-1.0] (p = 0.84)
Computer Access	0.98 [0.96 – 1.02] (p = 0.07)	1.01 [0.97-1.05] (p = 0.68)
Internet Access	0.98 [0.97 – 0.99] (p < 0.01)	0.97 [0.96-0.97] (p = 0.02)

**Fig. 1:**
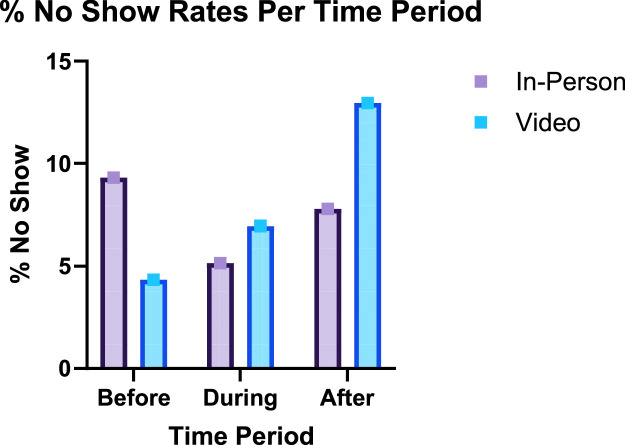
No show rates (%) by time-period: before, during, after comparing in-person versus video visits

Overall, no show rates for in-person visits were 8.48 % compared to 10.80 % for telemedicine visits. No-show rates for telemedicine visits during the pre-pandemic was 4.35 % versus 9.31 % for in person visits. (Fig. 1). During the pandemic, no show rates for in person visits dropped to 5.2 % while telemedicine rates increased to 6.9 %. After the pandemic, no show rates for both in person and telemedicine visits increased to 7.8 % and 12.9 %, respectively.

The most common diagnoses of patients seen by telemedicine and in-person were similar, regardless of time-period. During the pandemic, patients presented to in-person visits primarily for management of gastrostomy tubes (19.7 %), anorectal malformations (9 %) and umbilical hernias (3.4 %). Meanwhile, patients used telemedicine for anorectal malformations (6.4 %), umbilical hernias (5.3 %), and Hirschsprung's disease (4.1 %). Post-pandemic, patients presented most-commonly to in-person visits for gastrostomy tubes (8 %), umbilical hernias (6.2 %), and anorectal malformations (4 %). They continued to use telemedicine for anorectal malformations (6.2 %), umbilical hernias (4 %), and gastroschisis (3 %).

**Fig. 2:**
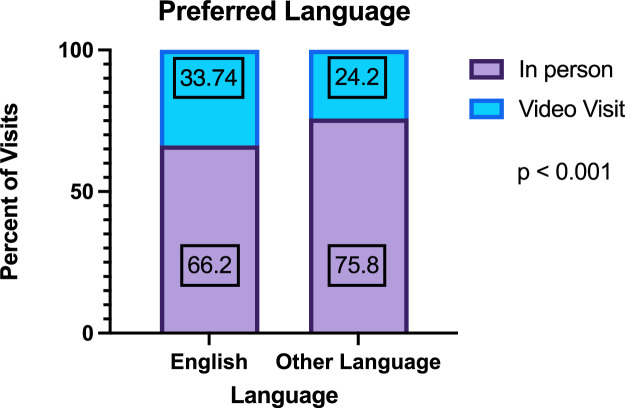
Percentage of visits in english and other language (including Spanish) for in-person or video visit.

The univariate mixed effect logistic regression model detailed in Table 1 showed that the odds of a telemedicine visit was 1.31 (CI 1.14-1.52, p < 0.01) for female compared to male patients. Older patients were more likely to use telemedicine by 1.6 % for every one-unit increase in age (OR 1.016, CI 1.0-1.03, p < 0.01). Preferred language by visit type is shown in Fig. 2. The odds of a telemedicine visit was 0.75 (CI 0.57-0.67, p < 0.05) for Spanish speakers compared to English speakers and 0.42 (CI 0.27-0.66, p < 0.01) for ‘other’ language speakers compared to English speakers. There was no significant difference in the odds of a telemedicine visit for those without private insurance compared to those with private insurance. Similarly, there was no significant difference in the odds of a telemedicine visit when comparing patients who listed their race as Asian, Black/African American, Hispanic/Latino, or Other compared to those who listed their race as White/Caucasian. However, those who listed their race as Unknown or declined to state had an odds of 1.43 (CI 1.1-1.9, p <0.05) of a telemedicine visit compared to those who listed their race as White/Caucasian (Table 1).

There was no significant difference in the odds of a telemedicine visit when comparing distance from the clinic. Using US census data, computer and internet access by zipcode was assessed. This data provided the probability of having a computer or the probability of having a computer based on zip code. The odds of having a telemedicine visit was 0.98 (CI 0.97 – 0.99, p < 0.01) as the probability of internet access in the patient's home zip code increased.

The multivariable mixed effect logistic regression that included all predictors of interest (thus controlling for confounding variables) showed that the variables identified in the univariate regression model still had a statistically significant effect on the type of visit.

The secondary analysis repeated the univariate and multivariable mixed effect logistic regression models to examine variables contributing to no show rates (Table 2). The univariate analysis showed that the odds of presenting to a scheduled telemedicine visit was 0.76 (CI 0.63-0.91, p-value < 0.01) compared to those who had an in-person visit. For telemedicine and in-person visits combined, the odds ratio of showing up was 0.66 (CI 0.543 - 0.806, p < 0.01) for those with public insurance compared to those with private insurance. When looking at self-identified demographics, the odds of presenting to a visit was 0.39 (CI 0.27 – 0.56, p < 0.01) for patients who identify as Black/African American, 0.65 (CI 0.51 -0.84, p < 0.01) for Hispanic patients, 0.78 (CI 0.57 – 1.08, p = 0.14) for those who identified as ‘other’, and 0.76 (CI 0.56 – 1.03, p =0.79) for those who identified as ‘unknown/declined’ compared to those who identified as White. There was a 2.12 (CI 1.17 – 3.82, p = 0.01) odds of coming to a visit for Asian patients compared to White patients. The odds of coming to a visit was 1.04 (CI 1.01 – 1.07, p < 0.01) for every one-unit increase in the probability of having computer access and 1.03 (CI 1.01 – 1.04, p < 0.01) higher for every one-unit increase in the probability of having internet access in the patient's zip code.

**Table 2:** Univariate mixed effect model: odds of completing a clinic visit by clinical variable. Multivariable mixed effect logistic regression analysis including all predictors of interest: odds of completing a clinic visit.

	Univariate Analysis: Odds of A Patient Attending a Clinic Visit [Confidence Interval] (p-value)	Multivariable Analysis: Odds of A Patient Attending a Clinic [Confidence Interval] (p-value)
Telemedicine		0.75 (CI 0.735-1.099 p <0.01)
Increasing Age	1.01 [0.99 – 1.03] (p = 0.44)	1.01 [1.11 – 1.63] (p < 0.01)
Female	0.91 [0.75 – 1.11] (p = 0.34)	0.89 [0.74 – 1.09] (p = 0.29)
Spanish Speaking	-	1.15 [0.79 – 1.69] (p = 0.46)
Other Language	-	0.77 [0.44 – 1.35] (p = 0.36)
Public Insurance	0.66 [0.54 – 0.81] (p < 0.01)	0.69 [0.56 – 0.85] (p < 0.01)
Race/Ethnicity		
Asian	2.12 [1.17 – 3.82] (p = 0.01)	2.11 [1.15 – 3.85] (p = 0.02)
Black or African American	0.39 [0.27 – 0.56] (p < 0.01)	0.43 [0.29 – 0.63] (p < 0.01)
Latinx/Hispanic	0.65 [0.51 -0.84] (p < 0.01)	0.70 [0.53 – 0.92] (p = 0.01)
Other	0.76 [0.56 – 1.03] (p =0.79)	0.78 [0.57 – 1.08] (p = 0.14)
Unknown/Declined	0.46 [0.31 – 0.68] (p < 0.01)	0.48 [0.33 – 0.73] (p < 0.01)
Distance From Clinic	1.00 [1.00 – 1.01] (p = 0.55)	1.00 [1.00 – 1.01] (p = 0.35)
Computer Access	1.04 [1.01 – 1.07] (p < 0.01)	0.98 [0.98 – 1.07] (p = 0.29)
Internet Access	1.03 [1.01 – 1.04] (p < 0.01)	0.98 [0.98 – 1.04] (p = 0.45)

Again, the multivariable mixed effect logistic regression that included all predictors of interest represented similar results. These results are details in Table 2. No significant difference in the odds of completing a visit was seen for age, sex, primary language spoken, distance from clinic, computer access, and internet access.

Lastly, a multivariable mixed effect logistic regression model adjusting for all the previous predictors as well as time-period, showed that the odds of presenting to a scheduled telemedicine visit was 0.54 (CI: 0.42 – 0.69, p-value < 0.01) compared to an in-person visit. Furthermore, the odds of presenting to a scheduled visit, telemedicine or in person, is 0.49 (CI: 0.36 – 0.66, p-value < 0.01) for those who had an appointment after the pandemic compared to those who had an appointment during the pandemic.

## Discussion

The rate of telemedicine utilization in this pediatric surgery clinic increased dramatically during the pandemic, then decreased after the pandemic. However, compared to pre-pandemic levels, there was still a 50-fold increase in telemedicine utilization. This suggests that the telemedicine infrastructure continued to be useful to patients and providers, even when the official stay-at-home order was lifted.

Barriers to telemedicine utilization for physicians have been largely alleviated by the state–of–emergency caused by the pandemic. Historically, pediatricians self-reported the largest barrier to offering telemedicine services to be inadequate reimbursement [4]. In 2010, reimbursement for telemedicine visits was only offered in 12 states, and this increased to 33 states and the District of Columbia in 2017 [10]. Only five states mandated payment parity with in-person visits [4]. The Supporting Pediatric Research on Outcomes and Utilization of Telehealth (SPROUT) Study in 2018 sought to assess the utilization of pediatric telehealth by distributing a survey to 56 programs across 30 states [10]. The survey identified the top four barriers for startup or growth for pediatric telehealth programs: reimbursement, lack of provider time, lack of provider interest, and state regulations. The removal of these barriers on the part of providers and the demands for adaptation mandated by the pandemic have resulted in robust telemedicine infrastructure that have facilitated the continued use of telemedicine post pandemic.

Increasing access to telemedicine visits may not necessarily increase access to care, however. This data shows that patients were more likely to no-show to their telemedicine visits than in-person visits. This is a surprising finding as telemedicine is thought to provide greater convenience to the patient/family. Before the pandemic, overall visits had a 9.26 % no-show compared to 10.21 % afterwards despite the increased telemedicine utilization in the post-pandemic period. In practice this amounts to only one more patient not showing up out of every 100. No-show rates varied more for telemedicine visits when comparing during pandemic to post-pandemic periods (6.95 % and 12.86 %, respectively).

Patients who identified as Black or African American were less likely to present to their visit compared to white patients, which correlates to telemedicine literature more broadly. One reason could be that telemedicine does not make visits accessible enough for this population. During the pandemic, African Americans and other communities of color (e.g. Indigenous, Lantinx, Asian) had a higher likelihood of being an essential worker, which could impact their ability to access care [7,11]. Similar studies in primary care found that audio-only visits are sometimes preferable for Black patients to give even more flexibility of location [11]. In our population, this would be difficult to coordinate as the child needs to be present at the visit, thus coordinating two sets of availability and travel. Another possible solution is to offer visits outside of work and school hours. Other barriers noted include confidence of the parent in using digital patient services [12]. One solution proposed in the literature to address this includes digital literacy readiness material to prepare and empower patients to navigate telemedicine spaces before setting up virtual appointments [12].

According to nationwide data, no-show rates improved post-pandemic compared to pre-pandemic numbers (pediatric clinic no-show rates of 17 %-19 %) [13,14,14]. No-show rates at our clinic have increased over time since the stay-at-home order ended, more drastically for telemedicine visits. This could be due to dissatisfaction with telemedicine services, frustration with language barriers, or a lack of perceived formality of the visit. It could also be due to technical difficulties at the time of a previous telemedicine visit leading to frustration and a no-show of the next visit. Simply increasing the availability of telemedicine post-pandemic is not sufficient for improving access to care for patients. Greater understanding of ongoing barriers is needed*.*

The first notable patient barrier is language. Previous studies at pediatric centers showed that during the pandemic, utilization among Spanish speaking patients dropped and patient satisfaction surveys showed less satisfaction than English speaking patients [15,16]. We found a statistically significant relationship between telemedicine utilization and language spoken by the patient: English-speaking patients were significantly more likely to utilize telemedicine encounters compared to patients who primarily speak non-English languages. From an institutional infrastructure standpoint, the availability of interpretation services, the range of languages available, and the ease of use for provider, patient and interpreter are essential for proper execution of a successful visit. Additionally, patient comfort with interpreting services and trust in the provider and healthcare system overall may contribute to whether they are open to completing a telemedicine appointment using an interpreter.

Other barriers to telemedicine include access to a stable and reliable internet connection, as well as digital literacy. Previous studies show 5 % of telemedicine visits could not be completed due to technical difficulties [1]. In our study, the association between internet access and choosing an in-person visit over a telemedicine visit was statistically significant but the OR of 0.98 likely has minimal clinical significance. On multivariate analysis, this correlation between internet access and completing a visit was not significant, indicating that internet access alone is not sufficient for completing effective interactions with the healthcare system. Access to the internet is helpful for scheduling and keeping track of clinic visits and results as many health systems are offering app-based digital patient charts. A patient must also have adequate digital literacy to navigate patient portals and instructions for downloading HIPPA-compliant video applications. Thus, further efforts to educate patients on how to use existing tele-health infrastructure may be needed to address such barriers.

Unexpectedly, distance from clinic did not impact telemedicine versus in-person preference. We also found that there were no clear diagnoses for which patients preferred an in-person visit. We expected that as the pandemic lifted, some families would opt for telemedicine visits due to distance or benign etiology. There are likely other factors that cause patients to prefer in-person regardless of distance from clinic, including acuity of care, type of specialty care, or need for physical exam for an initial visit. Insurance type (private versus government), another SDOH tied to health outcomes, did not predict the type of visit in our population. It seems that public insurance, including medical, does not prohibit a patient from choosing a virtual visit.

This study has multiple limitations. First, this is a single center experience and only looks at one pediatric subspeciality, pediatric surgery. This is a retrospective study, thus we can comment on correlation only. These findings may not be generalizable to other locations or other pediatric subspecialties. Additionally, the study lacks qualitative data such as patient satisfaction surveys or questionnaires to corroborate the quantitative data. Further studies using questionnaires would be helpful to parse apart patient barriers. Lastly, further analysis using different centers across the country would help give a broader picture of telemedicine utilization in the United States.

## Conclusion

This study demonstrates that the COVID-19 pandemic stay-at-home order drastically increased the use of telemedicine visits, even after the stay-at-home order ended. Despite increased access to telemedicine for patients and providers, there is still a higher no-show rate for telemedicine visits compared to in person visits. Further studies should focus on qualitative data from patients and family for preferences on visit type and reasons for failure for completion. Telemedicine is not without limitations, but we believe it has great potential for improving access to care especially for non-acute visit reasons and for individuals with limited ability to travel long distances to subspeciality appointments. Mitigating disparities in healthcare through telemedicine will require addressing SDOH-related disparities, particularly in language services for non-English speaking patients.

Supplement A: Example screenshot of publicly available Census Table using an example zip-code to show percent households with computer and internet access.

Category: Original Article

Previous Communication: Academic Surgical Congress Safety and Quality Meeting, Minneapolis, Minnesota, July 2023. National Medical Association Annual Meeting, New Orleans, Louisiana, August 2023

1. What is currently known about this topic?
  a. Telemedicine use expanded during the pandemic and infrastructure has remained widely available since.
2. What new information is contained in this article?
  a. Although telemedicine infrastructure remains, post-pandemic utilization of telemedicine for ambulatory pediatric specialty care has decreased. This may reflect patient and/or provider preferences and the limitations of telemedicine.
  b. Demographic differences exist in the use of telemedicine in the ambulatory pediatric specialty care setting. These differences vary before, during, and after state-mandated stay at home orders.

## CRediT authorship contribution statement

**Monalisa Attif Hassan:** Writing – original draft, Investigation, Data curation, Conceptualization. **Jeffrey Fine:** Formal analysis. **Kathleen Doyle:** Writing – review & editing. **Emily Byrd:** Writing – review & editing. **Minna M. Wieck:** Writing – review & editing, Visualization, Validation, Supervision, Resources, Methodology, Investigation, Conceptualization.

## Declaration of competing interest

The authors declare that they have no known competing financial interests or personal relationships that could have appeared to influence the work reported in this paper.

## Appendix Supplementary materials

Image, application 1
