# Supplementary material for: Disparities in the expansion of telemedicine in pediatric specialty care through the COVID-19 pandemic and beyond
Source: Surg Pract Sci. 2025 Feb 14;20:100275. doi: 10.1016/j.sipas.2025.100275 (PMC11871496; doi:10.1016/j.sipas.2025.100275)
Supplement: Supplementary file 1 [file mmc1.docx]

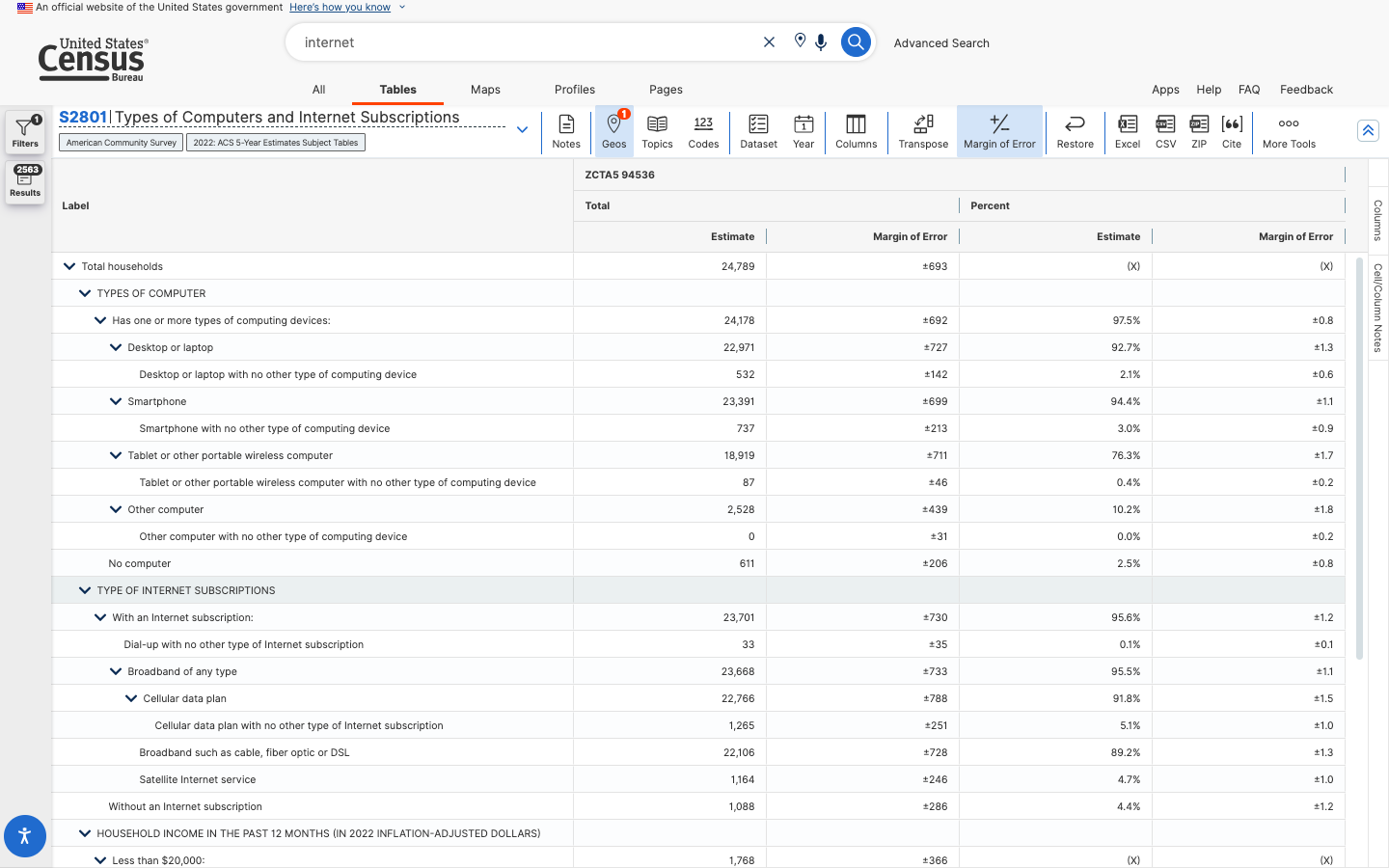


Supplement A: A representative screenshot using an example demographic zip code. This is done by searching ‘internet 94536’ on <https://data.census.gov/>. This leads to the above table (<https://data.census.gov/table/ACSST5Y2022.S2801?q=internet%2094536>). Percent estimates were used to approximate probability of having computer or internet access by patient zipcode.
